# Supplementary material for: Prepartum Vaccination Against Neonatal Calf Diarrhea and Its Effect on Mammary Health and Milk Yield of Dairy Cows: A Retrospective Study Addressing Non-Specific Effects of Vaccination
Source: Animals (Basel). 2025 Jan 14;15(2):203. doi: 10.3390/ani15020203 (PMC11758672; doi:10.3390/ani15020203)
Supplement: Supplementary file 1 [file animals-15-00203-s001.zip › File S1.pdf]

On-farm survey

Part I

<u>Vaccination management</u>

1. 1 Why did you decide to conduct the prepartum vaccination against neonatal calf diarrhea?

- ☐ Before the start of vaccination, problems with neonatal calf diarrhea occurred on the farm
- ☐ Before the start of vaccination, problems with other diseases occurred on the farm
- ☐ On the recommendation of the attending veterinarian
- ☐ Prophylaxis
- ☐ Other: ____

1. 2 Have you noticed any changes in your farm since the introduction of prepartum vaccinations neonatal calf diarrhea?

- ☐ No changes detected
- ☐ Yes, there have been <u>fewer</u> cases of neonatal calf diarrhea since then
- ☐ Yes, <u>fewer</u> diseases occur in calves
- ☐ Yes, <u>more</u> diseases occur in calves
- ☐ Yes, <u>fewer</u> diseases occur in dams
- ☐ Yes, <u>more</u> diseases occur in dams
- ☐ Other: ____

1. 3 Which maternity protection vaccine is used on your farm? Please enter the vaccines used and the corresponding period in the following table:

Period_(year)	Rotavec Corona (Intervet/MSD)	Lactovac C (Zoetis)	Scourguard 3 (Zoetis)	Bovigen Scour (Forte Healthcare)	Trivacton (Boehringer Ingelheim)	Other vaccine: __________ __________
From:<br>Until:						
From:<br>Until:						
From:<br>Until:						
From:<br>Until:						

1. 4 Do you apply the vaccination protocol from the instructions for use of the vaccine used?

- ☐ Yes
- ☐ No
- ☐ Not sure

1. 5 At what time are the vaccinations usually given? *(*some products are vaccinated once, some twice)*

- ☐ Single vaccination ______________weeks before expected calving date
- ☐ 1st vaccination ______________weeks before expected calving date
  2nd vaccination ______________weeks before expected calving date
- ☐ different vaccination schedule:____

1. 6 Which animals are included in the vaccinations?

- ☐ Cows
- ☐ Heifers (mature female cattle until the birth of the first calf)
- ☐ other animal groups, namely:
  ____

1. 7 Which vaccinations are carried out on your farm? *(*We are particularly interested in vaccines that are administered between 2 months before and 2 months after the expected calving date).*

- ☐ No
- ☐ Yes, namely the following (please write the name of the preparation in each case):
  - ☐ **Bronchopneumonia/calf flu**
    Name of the product: ____
    Group of animals: ____
    Administration: ________________________________Weeks *before calving*
    Administration: __________________________________Weeks *after calving*
  - ☐ **Trichophytosis/calf lichen**
    Name of the product: ____
    Group of animals: ____
    Administration: ________________________________Weeks *before calving*
    Administration: __________________________________Weeks *after calving*
  - ☐ **Clostridia**
    Name of the product: ____
    Group of animals: ____
    Administration: ________________________________Weeks *before calving*
    Administration: __________________________________Weeks *after calving*
  - ☐ **Mastitis (S. aureus, E. coli)**
    Name of the product: ____
    Group of animals: ____
    Administration: ________________________________Weeks *before calving*
    Administration: __________________________________Weeks *after calving*
  - ☐ **Coxiellosis/Q fever**
    Name of the product: ____
    Group of animals: ____
    Administration: ________________________________Weeks *before calving*
    Administration: __________________________________Weeks *after calving*
  - ☐ **Salmonella**
    Name of the product: ____

Group of animals: ____
Administration: ______________________________Weeks *before calving*
Administration: ________________________________Weeks *after calving*

- ☐ **Bovine viral diarrhea (BVD)**
  Name of the product: ____
  Group of animals: ____
  Administration: ______________________________Weeks *before calving*
  Administration: ________________________________Weeks *after calving*
- ☐ **Bluetongue disease**
  Name of the product: ____
  Group of animals: ____
  Administration: ______________________________Weeks *before calving*
  Administration: ________________________________Weeks *after calving*
- ☐ **Schmallenberg virus**
  Name of the product: ____
  Group of animals: ____
  Administration: ______________________________Weeks *before calving*
  Administration: ________________________________Weeks *after calving*
- ☐ **Bovine herpesvirus (BHV-1)**
  Name of the product: ____
  Group of animals: ____
  Administration: ______________________________Weeks *before calving*
  Administration: ________________________________Weeks *after calving*
- ☐ **Herd-specific vaccine against:** ___________________________
  Name of the product: ____
  Group of animals: ____
  Administration: ______________________________Weeks *before calving*
  Administration: ________________________________Weeks *after calving*
- ☐ **a vaccine not yet listed**
  Name of the product: ____
  Group of animals: ____
  Administration: ______________________________Weeks *before calving*
  Administration: ________________________________Weeks *after calving*

1. 8 Are other prophylactic measures (apart from vaccinations) carried out on the farm during the period around birth?

- ☐ No
- ☐ Yes, as follows:
  - ☐ Paramunity inducer (e.g., Zylexis®), at the following time: ___________________
  - ☐ Cytokines or chemokines (e.g., IL/interleukin-8 or Imrestor®), local or systemic, at the following time:____
  - ☐ Administration of immunoglobulins, at the following time:
    ____
  - ☐ Intramammary serum infusion (locally into the udder), at the following time:
    ____
  - ☐ Other: ____

Calf & colostrum management

2. 1 What quantity of colostrum is offered to the calves within the first 6 hours after birth?

- ☐ _______ liter(s)
- ☐ Ad libitum
- ☐ Unknown

2. 2 What is the average amount of colostrum drunk by calves in the first 6 hours after birth?

- ☐ ________ liter(s)
- ☐ Unknown

2. 3 If a calf does not consume colostrum within the first few hours after birth, how do you react?

- ☐ Drenching once with _______ liters of colostrum, at what point do you carry out this measure?
  ____
- ☐ Repeated drenching
- ☐ Other measure: ____
- ☐ No measure

2. 4 Is colostrum given beyond the calf's first day of life?

- ☐ No, colostrum is only given on the first day of life
- ☐ Yes, we administer (or mix) colostrum into the milk on the following days after birth
- ☐ Yes, with the following procedure: ____

2. 5 From which dams does the calf get colostrum?

- ☐ Colostrum exclusively from the calfs own mother
- ☐ Mixed colostrum
- ☐ Other: ____

2. 6. Is the colostrum treated before feeding?

- ☐ No treatment
- ☐ Pasteurization, heating to ____°C
- ☐ Colostrum is sometimes frozen
- ☐ Other: ____

2. 7. Which composition of milk do you choose?

- ☐ Milk replacer
- ☐ Whole milk
- ☐ Acidified milk
- ☐ Other: ____

2. 8. How much milk do you offer the calf in the first weeks of life?

- ☐ ______ Liter per day
- ☐ Ad libitum,
- ☐ Other: ____

Dry-off management

3. 1 How long is the dry period?

- ☐ Cows from the second lactation: _____ days (or ______ weeks)
- ☐ Cows lactating for the first time: _____days (or ______weeks)
- ☐ The dry period is individually adapted to the animal and lasts between __________ days and _____________ days

3. 2 How do you dry-off the animals?

- ☐ Selective drying-off (restricting the administration of antibiotic drying preparations to infected or diseased animals)
- ☐ Non-selective drying off (each cow is given an antibiotic drying off agent)
- ☐ Other: ____

3. 3 Do you use internal teat sealers?

- ☐ Yes, for all animals
- ☐ Yes, selective
- ☐ No

3. 4 Are the animals dried-off abruptly or gradually?

- ☐ Abrupt
- ☐ Gradual, by feed reduction
- ☐ Gradual, due to extended milking frequency
- ☐ Other: ____

Housing

4. 1 Please enter the housing type of the adult animals in the following table:

	Dry offs	Fresh milkers	Old milkers	Heifers*
Free stall barn				
Deep litter stable				
Tethering				
Access to the pasture				

*mature female cattle until the birth of the first calf

4. 2 How are the calves kept?

- ☐ in calf hutches
- ☐ in calf boxes
- ☐ Other: ____

4. 3 At what age are the calves transferred from individual housing to group housing?

With ________________ weeks of life

4.4 What is the average group size?

- ☐ < 6 animals
- ☐ 6 - 15 animals
- ☐ > 15 animals

4. 5 When do the calves leave your farm?

- ☐ Male calves on day __________
- ☐ Female calves on day __________
- ☐ As a rule, no female calf/young animal leaves the farm
- ☐ only the following female calves/young animals leave the farm: _________________________
- ☐ Other criteria: ____

Part II

Animal health management

Which of the following control measures do you take in the days around calving to detect diseases of the dam at an early stage or to prevent diseases?

	For all calvings	For unusual calvings	rarely/never	Unsure	Other
I observe the cow's general behavior					
I measure the body temperature with a thermometer					
I check the udder after calving					
I check whether the afterbirth was expelled					
I initiate measurements of ketone concentration (BHB) in milk, blood or urine					
The cow is given energy supplements as precaution (energy boluses, sodium propionate, propylene glycol, etc.)					
The cow is given calcium, phosphorus and/or glucose supplements as precaution					
The cow is given vitamin supplements and/or trace elements as precaution					
The cow is given vitamin D3 as precaution					
The cow is given monensin (e.g., Kexxtone) as precaution to prevent ketosis					

How is claw care carried out on the farm?

- ☐ Individual cows are cut if necessary
- ☐ Claw care is carried out in groups
- ☐ The entire herd is cut at once
- ☐ < 1x per year
- ☐ 1x per year
- ☐ 2x per year
- ☐ 3 times a year
- ☐ >3x per year

How often is lameness treated?

____
____
____
____
____

Birth monitoring

How is calving and birth monitoring carried out?

Where is the calving area located?

____
____
____

How are calving areas designed?

____
____
____

What is the hygienic condition of the calving area?

____
____
____

Which monitoring system is used?

____
____
____

How quickly is birth recognized?

____
____
____

When and how do you intervene in the event of birth problems?

____
____
____

Milk & Colostrum

Which milking system is used?

for the colostrum:

____
____

for the milk:

____
____

Which of the following are observed during milking?

On the animal:

- ☐ Pre-dipping
- ☐ Pre-milking
- ☐ Teat cleaning
- ☐ Post-dipping

How long is the preparation time?

____

General milking hygiene:

- ☐ Intermediate disinfection of the milking equipment between each cow
- ☐ Intermediate disinfection of the milking equipment between cow groups
- ☐ Complete cleaning and disinfection of the milking system after each milking cycle
- ☐ Spatial arrangement of the barn in order to encourage cows not to lie down directly after milking, but to eat (e.g., distance between lying area and exit of the milking area)
- ☐ Hand washing and disinfection facilities available for milkers

Other: ____

Are milk samples tested for pathogens?

Which indicators justify an investigation for mastitis pathogens?

____
____
____

Own laboratory, or outside?

____
____
____

Which pathogens are predominant in the farm?

____
____
____

Do you carry out quality examination of colostrum?

- ☐ No
- ☐ Yes, with a manual refractometer (Brix)
- ☐ Yes, with a digital refractometer (Brix)
- ☐ Yes, with a spindle
- ☐ Yes, determination of blood IgG in the laboratory
- ☐ Yes, with the following system:
  ____
- ☐ Results of the quality examinations:
  ____
  ____
  ____

Stable hygiene

Examination of the degree of hygiene of the animals (e.g., random sampling of 20 animals)

Tier ID .	Grade 1	Grade 2	Grade 3	Grade 4	Tier ID .	Grade 1	Grade 2	Grade 3	Grade 4

*Use of the hygiene score, consideration of the degree of soiling of limbs, udder and flank

What hygiene measures are carried out before newborn calves are housed?

- ☐ Cleaning
- ☐ Disinfection
- ☐ Vacancy
- ☐ Moving hutches
- ☐ Other: ____

Is the ingredient halofuginone used in hygiene products?

- ☐ No
- ☐ Yes, as recommended in the instructions for use
- ☐ Another active substance against cryptosporidia, namely: ___________

Feeding

Do you record the body condition score?

How frequently and regularly is it recorded?

____
____
____

What influence does this have on feeding management?

____
____
____

What type of ration is fed?

- ☐ TMR (total mixed ration)
- ☐ AMR (enhanced mixed ration - partial TMR with individual concentrate allocation)
- ☐ Other: ____

How is feeding of dry-offs organized?

- ☐ Single phase (uniform feeding for all dry-offs)
- ☐ Two phases (division into two groups of dry cows with different rations), if yes, how many weeks before calving do the animals enter the transit group?
  ________ weeks
- ☐ Other: ____

How are the lactating cows fed?

- ☐ Single phase (uniform feeding for all lactating cows)
- ☐ Two phases, divided into the following yield groups:
  ____
  ____
- ☐ Other: ____

Please describe the method of weaning the calves?

____
____
____
____
____

Calf husbandry

Assessment of the climatic conditions

- ☐ Outdoor climate
  - for the following (age) groups: ____
- ☐ Indoor climate
  - for the following (age) groups: ____
- ☐ Temperature
  - too cold
  - appropriate
  - too warm
- ☐ Pungent ammonia odor
- ☐ other notes:

  ____
  ____
  ____
